# Glycerine in Phthisis

**Published:** 1857-02

**Authors:** 


*Glycerine in Phthisis.*

Some other correspondents have written, requesting us to state *how* we use the glycerine in the treatment of consumption; and as it is almost impossible to find time to answer all the letters addressed to us, we will give the following formulæ. For cases of tubercular disease in its early stage, before the cough is accompanied by much expectoration, we more frequently prescribe:—

℞—Glycerine, . . . . ℥ij.

Iodide of Potassa, . . ʒj.

Sulph. of Morphine, . 2grs.

Mix,

And give one tea-spoonful before each meal and at bed-time.

If the disease is farther advanced, and expectoration more copious, with rapidly-increasing emaciation, we prefer the following:—

℞—Glycerine, . . . ℥ij.
Syrup of Iodide of Iron, ℥ss.
Sulph. Morphine, . . 2grs.
Mix,

And give one tea-spoonful every four or six hours.

It is now two years since we commenced using the glycerine in the treatment of phthisis, generally combining it with some preparation of iodine, and just enough morphine to allay cough and promote rest; and we have certainly derived more benefit from it than from any other one remedy.

